# Applications of Nanosheets in Frontier Cellular Research

**DOI:** 10.3390/nano8070519

**Published:** 2018-07-12

**Authors:** Wenjing Huang, Yuta Sunami, Hiroshi Kimura, Sheng Zhang

**Affiliations:** 1 Micro/Nano Technology Center, Tokai University, 4-1-1 Kitakaname, Hiratsuka-city, Kanagawa 259-1292, Japan; huang@tsc.u-tokai.ac.jp (W.H.); hkimura@tokai-u.jp (H.K.); 2 Department of Bioengineering, School of Engineering, The University of Tokyo, 7-3-1 Hongo, Bunkyo-ku, Tokyo 113-8656, Japan; 3 Department of Mechanical Engineering, Tokai University, 4-1-1 Kitakaname, Hiratsuka-city, Kanagawa 259-1292, Japan; 4 Division of Biomedical Engineering, Renal Division, Department of Medicine, Brigham and Women’s Hospital, Harvard Medical School, Cambridge, MA 02139, USA

**Keywords:** nanosheet, cell adhesion, drug delivery, cell capturing, regenerative medicine

## Abstract

Several types of nanosheets, such as graphene oxide (GO) nanosheet, molybdenum disulfide (MoS_2_) and poly(l-lactic acid) (PLLA) nanosheets, have been developed and applied in vitro in cellular research over the past decade. Scientists have used nanosheet properties, such as ease of modification and flexibility, to develop new cell/protein sensing/imaging techniques and achieve regulation of specific cell functions. This review is divided into three main parts based on the application being examined: nanosheets as a substrate, nanosheets as a sensitive surface, and nanosheets in regenerative medicine. Furthermore, the applications of nanosheets are discussed, with two subsections in each section, based on their effects on cells and molecules. Finally, the application prospects of nanosheets in cellular research are summarized.

## 1. Introduction

To date, many fabrication technologies have been developed for different types of nanosheets, such as graphene oxide (GO) nanosheet, molybdenum disulfide (MoS_2_), and poly(l-lactic acid) (PLLA) nanosheets [1,2,3,4]. Shortly after the development of the fabrication technology, researchers applied nanosheets to cellular research. Monocrystalline graphitic films a few atoms thick were first reported in 2004, and MoS_2_ nanosheets were fabricated successfully in 2011 (Liu et al., 2008) [2]. Furthermore, NO nanosheets were applied as carriers of SN38 in 2008—a drug with high cancer cell killing potency—and MoS_2_ nanosheets were used for the detection of DNA and small molecules due to their fluorescence-quenching ability and DNA affinity [5,6,7]. Fujie et al. developed fabrication techniques for PLLA nanosheets in 2007, and they investigated the cell adhesion properties of the sheets in 2011 [3,8]. As is known, most nanosheets are transparent and have excellent electrical and thermal properties with a large size-aspect ratio (>10^6^). Therefore, the application of nanosheets in relation to cellular research is due to the following properties. (1) The nanosheet surface can be modified (biofunctionalized) relatively easy for applications in cellular research [9,10,11]. (2) Cells or molecules show functional responses after the interaction with materials of nanosheets [12,13,14]. (3) Nanosheets are flexible, and cells adhered to the sheets are transplanted without cell detachment [15]. Further, fragments of nanosheets can be endocytosed by cells [16,17].

Besides GO, MoS_2_, and PLLA, many other nanosheets or two-dimensional (2D) materials have been newly developed. For example, in 2017, π-conjugated 2D porous organic nanosheets called NUS-24 were developed, and they showed high sensitivity toward Fe^3+^ ions and nitro-containing compounds, although their application in cellular research has not been realized [18]. On the other hand, many of the 2D graphene analogues, including π-conjugated 2D porous organic nanosheets, 2D graphene analogues (such as hexagonal boron nitride (h-BN), carbon nitride, and transition metal di-chalcogenides), have been developed, and some of them were applied in cellular research [19,20,21]. Analogues, such as h-BN and monolayer 2D graphitic carbon nitride (g-C_3_N_4_) nanosheets, were fabricated and used for cell imaging [19,22]. Further, there are techniques to prepare nanosheets on titanium surfaces for applications in biomedical research [13,23,24]. Therefore, nanosheets made from various types of materials have been used in cellular research, which makes their application extensive.

Diverse research on the application of nanosheets promotes the development of new technologies for disease diagnosis/therapy and regenerative medicine at cellular levels [25,26,27,28,29]. This review focuses on the research of cell capturing, regulation of cell adhesion and function, protein delivery into cells/transfection, cellular-level sensing/imaging, and regeneration regulation at cell levels using nanosheets.

## 2. Nanosheets as a Substrate

### 2.1. Effects of Nanosheets on Cell Adhesion and Function

**Figure 1:**
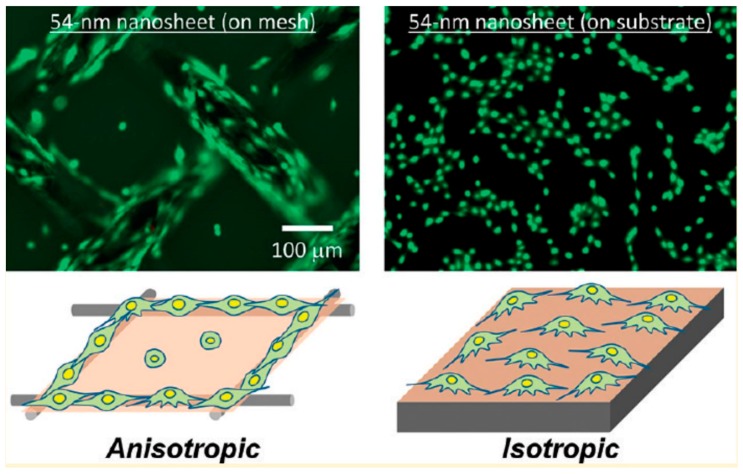
Substrata effect on nano–bio interface [8]. PLLA nanosheets were placed onto a metal substrate or a mesh to evaluate the effects of substrate stiffness on cell adhesion. Most of the H9c2 cells adhered onto the nanosheet part of the metal wire but not the part of the meshed lattice. On the other hand, cells adhered almost evenly across the substrate without differences in stiffness. Reproduced from [8] with permission from American Chemical Society, 2018.

Cell adhesion to nanosheets with various functions was investigated for further applications. Cells adhere to the extracellular matrix (ECM) through transmembrane proteins called integrins, which are a type of focal adhesion protein that serve as the mechanical linkages to the ECM. Substrate conditions, such as coating of extracellular matrix, substrate stiffness, and substrate topology, are important for cell adhesion and growth. A study by Okada et al. suggested that hydroxyapatite (HAp) nanosheet substrates with contact domains larger than 100 nm were required for the stable adhesion of rat bone marrow-derived mesenchymal stem cells [30]. Da Silva et al. developed an ultrathin flat sheet with controlled wettability using the spontaneous assembly of a peptide bolaamphiphile called RFL_4_FR (R, arginine; F, phenylalanine; L, leucine). Growth of human corneal stromal fibroblast (hCSF) cells was successful on this type of nanosheet, due to the wettability of the bolaamphiphile peptides resulting from hydrophilic peptide groups at both ends of a long hydrophobic hydrocarbon chain or peptide sequence [31]. The adhesion properties of nanosheets can be modified by the coating of ECM, and the coating method may influence the cell adhesion properties. Poly-l-lactic acid (PLLA) nanosheets possess anti-adhesive properties and can prevent unwanted wound adhesion, therefore, the nanosheets can be used as wound dressing. Niwa et al. showed that spin-coating collagen on the PLLA nanosheet promotes the adhesion of murine fibroblast cell line NIH3T3 [32]. Cell adhesion is also dependent on the stiffness of the underlying substrate: when rat cardiomyocytes were cultured on PLLA nanosheets placed on flat SiO_2_ substrate and metal meshes, cells showed almost equal distribution on the substrate but adhered specifically to the parts along the metal meshes (Figure 1) [8]. In addition, some novel types of nanosheets are developed specifically for cell culture and tissue engineering. Laurenti et al. developed a magnesium phosphate nanosheet-based thixotropic gel, and osteoblast cells could adhere onto the 2D structures and form colonies [33]. Functionalized nanosheets were also synthesized onto traditional medical materials for improving cell culture. For example, when calcium-containing nanosheets were synthesized onto a sandblasted and acid-etched (SLA) titanium surface, a sustained release of Ca^2+^ ions was measured, and adhesion/spreading of MC3T3-E1 cells was upregulated [34]. Coating of flower-like calcium phosphate nanosheets onto a titanium surface can also promote cell adhesion and spreading due to its wettability [35].

**Figure 2:**
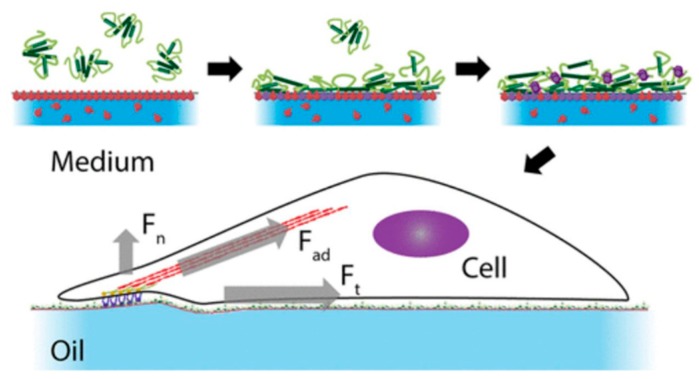
Schematic figure of a cell applying forces across an oil–water interface in the normal and tangential directions. Cell spreading and proliferation are enabled by the protein nanosheets [36]. Forces are presumed to be transmitted from the cell cytoskeleton to the protein nanosheets (extracellular environment) through focal adhesion proteins, and the forces exerted by cells are thought to be counterbalanced by the strength of the protein nanosheets. Reproduced from [8] with permission from American Chemical Society, 2018.

The adhesion of HaCaT cells—a spontaneously transformed aneuploidy immortal keratinocyte cell line—was realized on the surface of low-viscosity liquids (for example, a fluorinated oil (Novec 7500, 0.77 cSt) containing the surfactant) by the self-assembly of mechanically strong protein nanosheets on a liquid surface (Figure 2) [36]. Cell adhesion and spreading are critical for various cell functions, and enhancing the adhesion of rat bone marrow cells by culturing on nanosheets of titanium alloys resulted in increased osteogenic differentiation [13]. Nanosheets, such as graphene oxide (GO) nanosheets, are used extensively in living systems. Even so, we cannot ignore some toxic effects of nanosheets on cells. Although this type of nanosheet is not cytotoxic, RBL-2H3 cells cultured on the nanosheet may “feel” environment stress, and shedding of plasma membranes (e.g., detection of membrane fragments) was confirmed [37]. Further, disruption of the cell cytoskeleton was observed in A549 lung carcinoma cells cultured on GO nanosheets [38].

Pan et al. fabricated positively or negatively charged palladium nanosheets by treating the sheets with short chain thiolated carboxylic acids or amines, and they reported that cytotoxic effects may depend on the surface charge. 80% of HepG2 cells died on the positively charged nanosheets, however, only 30% died on the negatively charge nanosheets [39]. Molybdenum disulfide (MoS_2_) nanosheets were also reported to disrupt structures of α–helical peptides, a common motif in the secondary structure of proteins [40]. On the other hand, scientists are making great efforts to reduce the cytotoxicity. Sasidharan et al. reported that cell apoptosis was observed on as-synthesized graphene because of high oxidative stress, but no toxicity was observed on carboxyl hydrophilic graphene [41]. Likewise, graphene modified with biocompatible polyethylene glycol (PEG) did not show acute toxicity [11,42]. PEGylation decreased the affinity of graphene-based nanomaterials, so, coating nanomaterials with PEG chains was reported as a method to reduce toxicity [43,44]. However, we note that Luo et al. demonstrated that PEGylation could not bypass cytotoxicity, because interactions between PEGylated GO and the membranes of macrophage cells promoted high levels of cytokine secretion through integrin-mediated signaling pathways [45]. As an alternative, GO was functionalized with poly(acrylic acid), and the in vitro and in vivo biocompatibility was found to be superior to PEGylation. The differences in biocompatibility may result from differential compositions of protein corona formed on the surface of GO, because the composition may influence their interaction with cell membrane and cellular uptake [46].

### 2.2. Delivery Functions Acted on by Nanosheets

**Figure 3:**
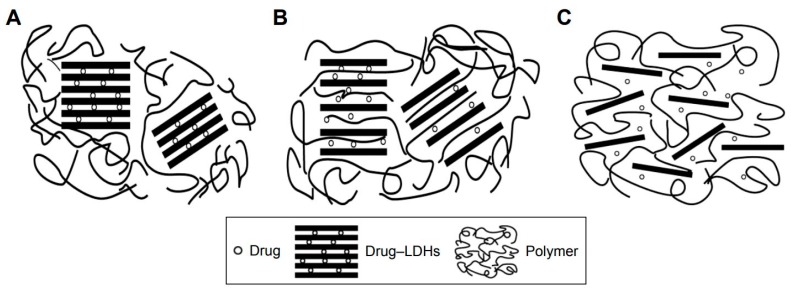
Schematic diagrams of (**A**) organic-coated inorganic hybrid layered double hydroxide (LDH) nanoparticles; (**B**) organic-intercalated inorganic hybrid LDH nanoparticles; and (**C**) organic–inorganic hybrid LDH nanosheets [50]. Reproduced from [50] with permission from Dove Medical Press, 2018.

Various types of nanosheets provide flexible platforms for cell culturing, and the surface-modified nanosheets exhibit excellent drug- or DNA-binding properties. Therefore, nanosheets are naturally used as tools in the delivery of drugs, cell monolayers, and genes. Fujie et al. developed a micropatterned and biodegradable poly(lactic-*co*-glycolic acid) (PLGA) nanosheet for the delivery of engineered epithelial monolayers into the narrow subretinal space by a simple injection through a syringe needle [25]. Nanosheets with adhered retinal pigmented epithelial cells can withstand deformation in a syringe needle without heavily damaging the cells [25]. This method is impossible for conventional delivery methods, which have used collagen, poly(ethylene terephthalate) (PET), or poly(methyl methacrylate) (PMMA) with sizes of several micrometers to several millimeters as delivery substrates [47,48,49]. Various structures developed from nanosheets were applied as drug carriers. For example, chitosan-glutathione-valine-valine layered double hydroxides (LDHs) were used to deliver drug to human corneal epithelial primary cells and retinal pigment epithelial cells, and nanosheets with drug (pirenoxine sodium) were uptaken through a cell membrane transporter, peptide transporter-1 (Figure 3) [50]. GO nanosheets were modified by natural peptide protamine sulfate and sodium alginate, and self-assembly of the nanosheets was developed as a drug delivery system. The system possessed properties such as a pH-sensitive release of anticancer drugs and suppression of protein adhesion. Further, the modified GO nanosheets could be uptaken by MCF-7 cells (breast cancer cells) [51]. Delaminated Mg-Al-lactate layered double hydroxide nanosheets were used as vectors to deliver salmon DNA to 293T cells, which was facilitated by the sheets’ high DNA adsorption capacity and ability to be uptaken by cells [10]. Black phosphorus (BP) nanosheets were modified with polyethylene glycol amine to improve the loading capacity of theranostic agents such as doxorubicin (DOX) for chemotherapy and cyanine7 (Cy7) for in vivo near-infrared (NIR) imaging [52]. Greek Testudo-like structures were developed from two-dimensional MoS_2_ nanosheets with DNA oligonucleotides, and the structures responded to heightened ATP metabolism in cancer cells and to released drugs [53].

Conventional transfection processes for cell transfection or gene therapy are complicated, toxic, and less effective, therefore, nanosheets were used as advanced tools for nonviral-based gene delivery substrates. When GO nanosheets formed a complex with cell-penetrating peptides (CPPs), the cytotoxicity of CPPs was mitigated and the transfection efficiency of CPPs with oligonucleotides was highly improved [54]. Positively charged MoS_2_-polyethylene glycol-polyethylenimine nanosheets were loaded with small interfering RNA and added to the cell (HepG2 cell line) culture medium for cell transfection [16]. Unlike the method of adding nanosheets to the cell culture medium (solution-based transfection), Ji et al. developed a silica nanosheet to immobilize the DNA complex, and transfected the cells attached to the nanosheet. The transfection efficiency was approximately 20% higher than that of solution-based transfection [55]. Further, naked DNA on silica glass without any vectors was transferred into human mesenchymal stem cells, a difficult-to-transfect cell type [56].

## 3. Nanosheets as a Sensitive Surface

### 3.1. Cell Capturing Using Surface-Modified Nanosheets

**Figure 4:**
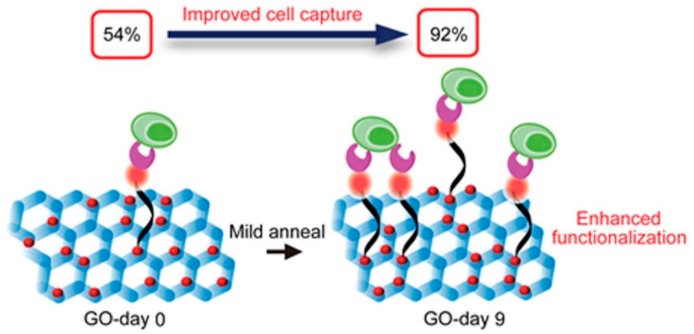
Cell capture improvement by using graphene oxide (GO) nanosheets through oxygen clustering [63]. A mild thermal annealing treatment was used to control and tune the distribution of oxygen functional groups on GO. Reproduced from [63] with permission from American Chemical Society, 2018.

Several technologies have been developed for the capturing of circulating tumor cells, such as microfluidic chips and functionalized and structured medical wire [57,58,59,60,61]. Even so, nanosheets together with other nanomaterials have emerged as effective tools for the isolation of tumor cells [62]. Surface modification of graphene oxide (GO) nanosheets can be easily realized, and GO nanosheets coated with specific antibodies were used as tools for cell capturing. GO nanosheets are carbon-based materials with a benzene basal structure [1]. The structure can interact with the aromatic functional groups of most biomolecules of interest [63]. Since a cell capturing system using three-dimensional microfluidic devices limits cell spreading and further observation on the chip after capturing, Yoon et al. developed GO nanosheet platforms absorbed onto arrayed flat (two-dimensional) gold patterns. The nanosheets were coated with NeutrAvidin, and two human breast cancer cell lines (MCF-7 and Hs-578T) and a human prostate cancer cell line (PC-3) were captured by the interactions between NeutrAvidin and biotinylated EpCAM antibody [64]. In the study by Chen et al., GO nanosheets were coated with single-domain antibody fragments for two different types of white blood cells [65]. Instead of using as-synthesized GO nanosheets, Bardhan et al. improved the nanosheets’ function through changing the distribution of oxygen functional groups before coating with an antibody fragment (VHH7), and Class-II MHC-positive cells from murine whole blood were captured quickly and effectively (Figure 4) [63].

### 3.2. Cell Sensing and Imaging

**Figure 5:**
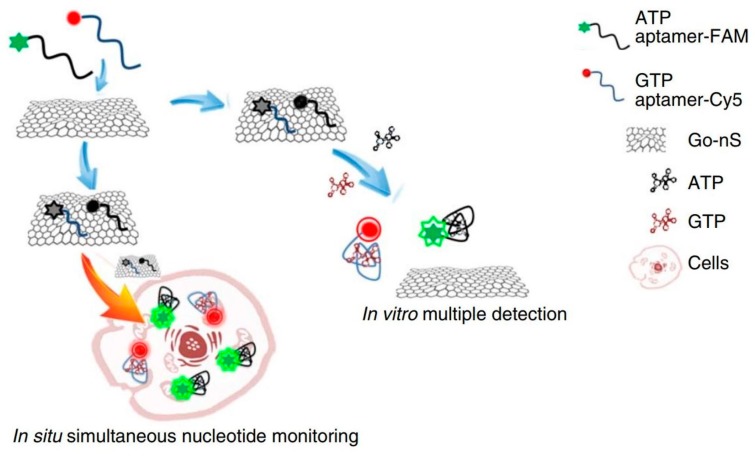
Schematic figure of in vivo and in situ molecular probing in living cells by using the aptamer/GO-nanosheet nanocomplex [79]. When ATP aptamer–FAM and GTP aptamer–Cy5 bind to GO nanosheets, fluorescence is “off” because of the fluorescence-quenching ability of GO. On the other hand, fluorescence is “on” when the aptamers are released into cells. Reproduced from [79] with permission from Nature Publishing Group, 2018.

Nanosheets are used extensively in the fields of sensing and imaging related to cellular research [9,10,22,26,66,67,68,69,70,71]. Cell-signaling sensing using nanosheets will shed new light on essential cell/protein functions. For example, hydrogen peroxide (H_2_O_2_) signaling in cells is important, because it is involved in diseases such as Alzheimer’s, Parkinson’s, and cancer [72,73,74,75]. MoS_2_ nanosheets enable the sensing of cell H_2_O_2_ signaling due to their edge sites displaying electrochemical activities [76]. Based on this understanding, Tang et al. developed a three-dimensional nanosheet for the effective sensing of H_2_O_2_ in living RAW 264.7 macrophage cells and neurons on tissue-like three-dimensional structures by exposing abundant nanosheet edge sites [77]. Cytokine tumor necrosis factor (TNF)-α in live cells can be detected by a reduced GO decorated with gold nanoparticles and aryldiazonium salts [78]. A manganese dioxide (MnO_2_) nanosheet-aptamer nanoprobe was fabricated for tumor cell imaging based on a dual-activatable fluorescence/MRI strategy, and the advantages are as follows: (1) Fluorescence signals (aptamers, fluorescein amidate-labeled 7-base DNA) was quenched on the MnO_2_ nanosheets. After the nanosheets were uptaken by tumor cells, the adsorption of aptamers became weak, and partial fluorescence was recovered; (2) The MnO_2_ nanosheet outside the tumor cells has low T_1_- or T_2_-weighted contrast, and after the nanosheets were uptaken by tumor cells, large amounts of Mn^2+^ ions were generated for MRI imaging [17]. Similarly, using the fluorescence-quenching ability of GO nanosheets, a platform for the detection of multiple nucleotides was created: the fluorescence was turned “off” by ATP aptamer–FAM and GTP aptamer–Cy5 binding to the GO nanosheet; aptamers were released into cells after the detection of target nucleotides, leading to fluorescence being turned on (fluorescence off/on switch) (Figure 5) [79].

## 4. Nanosheets in Regenerative Medicine

### 4.1. Nanosheets as a Scaffold Element

As a scaffold element for tissue regeneration, nanosheets show excellent application features.

**Figure 6:**
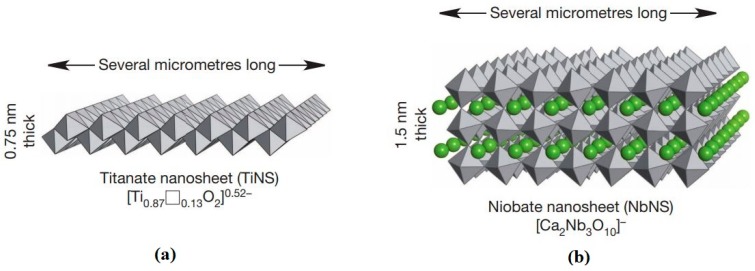
Schematic figure of negatively charged (**a**) unilamellar metal oxide nanosheets of titanate (TiNS) and (**b**) niobate (NbNS) [80]. The cofacially oriented charged nanosheets embedded in hydrogels made the material deform easily when exposed to shear forces while being resilient to compressive forces in the vertical direction. Reproduced from [80] with permission from Nature Publishing Group, 2018.

Firstly, nanosheets were used to construct three-dimensional structures and endowed the structure with tissue-like anisotropic mechanical properties. A three-dimensional mechanical microenvironment is important for cell differentiation and growth. Liu et al. built up a hydrogel structure with a titanate(IV) nanosheet, and the structure possessed the same anisotropic mechanical properties as articular cartilage. Titanate nanosheets in aqueous colloidal dispersions were exposed to a strong magnetic field, and the nanosheets aligned cofacially. The anisotropic hydrogel structure can withstand compressive forces in the vertical direction, but it is deformable in the horizontal direction (Figure 6) [80]. Nanosheets were used to improve the mechanical properties of scaffolds for cell culturing [81]. For example, Shuai et al. reported that induction of boron nitride nanosheets enhanced the compressive stress of an akermanite scaffold, a promising bioactive material in the field of tissue engineering. In addition, the seeded MG63 osteoblast-like cells and human bone marrow stromal cells adhered and proliferated well [82]. Further, a MoS_2_-akermanite three-dimensional scaffold for tissue regeneration after the removal of tumor tissues was developed using a three-dimensional printing technique [83].

Secondly, nanosheets are applied as two-dimensional cell sheets. Cell sheets mimic basement membranes. In vivo, the membrane is composed of proteins such as type-IV collagen, laminin, and fibronectin, and has nano-/micropores for cell–cell communication. A porous nanosheets made from a polymer ethyl acetate solution consisting of poly(d,l-lactic acid) and polystyrene. Using the nanosheets, a multilayered structure was created by rolling the nanosheets with adherent C2C12 myoblasts around a cylinder-shaped template [28].

Thirdly, nanosheets are used as a cell carrier for transplantation into a narrow space. As stated by Suzuki et al., nanosheets, such as a self-assembled monolayer (SAM) of thiol molecules, can be used as a cell carrier without changing the cell–cell contacts during the process of transplantation. A problem is that the SAM is adsorbed onto a gold surface, and scientists have to consider the harvesting method. A negative electrical potential was applied to make the nanosheet with cells to disconnect from the underlying gold surface for transplantation [15].

### 4.2. Nanosheets for Stem Cell Differentiation and Tissue Regeneration

**Figure 7:**
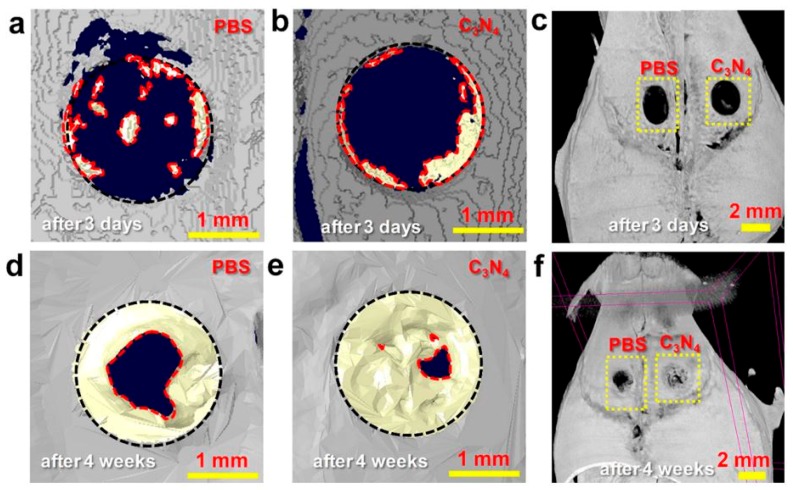
(**a**–**f**) Three-dimensional μ-CT images after 3 days or 4 weeks of PBS and C_3_N_4_ nanosheet-assisted treatment for the enhanced repair of cranial bone defect under red light in vivo [88]. C_3_N_4_ sheets showed the potential for promoting bone formation. Reproduced from [88] with permission from American Chemical Society, 2018.

Various types of nanosheets have been used for cell differentiation and tissue regeneration at protein and cellular levels. Nanosheets were used in several studies to control the differentiation of stem cells into the desired cell types (the control of stem cell fate), and these studies were reviewed in an article by Kenry et al. [29]. A specific physical and topographical microenvironment resulting from a nanosheet-based platform may regulate stem cell differentiation [84]. GO nanosheets were reported to sustain the self-renewal of mouse embryonic stem cells via a signaling pathway involving integrin [85]. Graphene can also be used to induce the differentiation of mesenchymal stem cells and neural stem cells [86]. Due to the high affinity of insulin resulting from H-bonding and electrostatic interactions, the culture of bone marrow-derived mesenchymal stem cells (MSCs) on GO nanosheets led to adipogenesis [87]. With regard to cell-based tissue regeneration, carbon nitride (C_3_N_4_) nanosheets were used in bone regeneration with the following properties: (1) This carbon nanosheet had a size of less than 200 nm and low toxicity; (2) The nanosheet can disperse in water easily due to structural polarization (polarized C and N atoms) from positive or negative charges. Red light induced a transient photocurrent of C_3_N_4_ nanosheets, and the photocurrent led to the translocation of Ca^2+^ and increase in cytosolic Ca^2+^. Red light absorption of the sheets resulted in human bone marrow-derived mesenchymal stem cells (Figure 7) [88].

## 5. Important Issues: Internalization, Distribution, and Cellular Responses

The above-mentioned studies have shown that cells are capable of internalizing nanosheets, and the internalization and cellular distribution is related not only to the cytotoxicity of nanosheets but also to their potential applications. There are several mechanisms of eukaryotic endocytosis (cell transport of exogenous molecules into the cell), such as clathrin-mediated and caveolae-mediated internalization and macropinocytosis, and the endocytotic mechanism “selected” by cells may depend on the size of the exogenous molecules [89]. The internalization mechanism may depend on types of cells and nanosheets, and the distribution of uptaken nanosheets throughout the cell membrane was observed in several organelles. In a study, fluorescein isothiocyanate (FITC)–GO material (PEGylated GO) was added to the culture medium of Sao-2 osteoblasts, MC3T3-E1 preosteoblasts, and RAW 264.7 macrophages seeded on glass coverslips to observe its internalization and distribution. Results showed that internalization of nanosheets depended on the cell type, and FITC–GO molecules were co-localized with F-actin filaments and induced cell-cycle alterations, apoptosis, and oxidative stress [90]. Macropinocytosis was found to be the general mechanism for internalizing FITC-PEG-GOs (ca. 100 nm) for human Saos-2 osteoblasts, human HepG2 hepatocytes, and murine RAW 264.7 macrophages, although there may be additional endocytotic mechanism (e.g., clathrin-dependent mechanisms and phagocytosis) for each cell type [91]. Protein (FITC–BSA)-coated GO nanosheets entered cells after surface adhesion for 30 min and appeared in intracellular vesicles [92]. The internalization processes of few-layer pristine graphene (GR) and monolayer GO flakes by primary cortical neurons were observed in a study by Bramini et al. Exposure to GO, but not GR, reduced the number of excitatory synaptic contacts and altered the Ca^2+^ dynamics, and a high percentage of GO flakes were found to enter the lysosome [93]. Similarly, a dual-modal stimulated Raman scattering (SRS)/transient absorption (TA) microscopy technique was developed to observe the distribution of MoS_2_ nanosheets in live HeLa cells, and it was observed that MoS_2_ localized with lipid-rich structures, such as endosomes and lysosomes [94]. Further, the nanoassembly of GO/poly(sodium-4-styrenesulfonate) (PLL)/PEGylated PLL can cross the cell membrane, after which it localizes in the lysosomal compartment. Moreover, GO and TiO_2_–GO composites entered A549 cells and localized to the cytoplasm and nucleus [95,96].

As demonstrated by Zhand et al. in a paper discussing the interaction between mammalian cells and graphene materials, the internalization of nanosheets may alter the distribution of intercellular organelles [97]. For example, redistribution of cytoplasmic lactate dehydrogenase (LDH) after exposure to GO and redistribution of mitochondrial cytochrome c in RAW 264.7 cells cultured in a medium with pristine graphene were observed in experiments [98,99]. An interesting observation is that normal and cancer cells may respond differently to nanosheet internalization, and nanosheets have been applied as theranostic materials based on their specific biodistribution and release at the tumor sites and distribution of nanosheets in cancer cells [100,101]. Zhou et al. reported that mitochondrial oxidative phosphorylation (OXPHOS) was impaired in breast cancer cells (MDA-MB-231, MDA-MB-436, and SK-BR-3) by PEG–GO, but OXPHOS in noncancerous cells (MCF-10A) was almost unchanged. A phenomenon like this may be related to the development of a new approach to treating breast cancer [102]. Moreover, compared to normal tissues, tumor tissues have an acidic environment (pH value ranging from 4.5 to 5.0), and MnO_2_ nanosheets functionalized with hyaluronic acid (HA) were used to deliver cisplatin (cis-diamminedichloroplatinum (CDDP)) to tumor cells in tumor-bearing animals (mice). These nanosheet-based drug carriers were observed to specifically distribute in the cytoplasm of A549 cancer cells after intracellular uptake and enhance apoptosis of tumor cells [103,104]. GO nanosheets may not be effective for the delivery of anticancer drugs, but polyethylene glycol-grafted GO (pGO) combining doxorubicin (DOX) with the photosensitizer chlorin e6 (Ce6) could accumulate in tumor sites over 3 days. This nanosheet-based carrier was distributed in the cytoplasm of SCC7 cells, and disruption of tumor nuclei was observed in tumor sections [105]. α_v_β_3_ integrin was overexpressed on cancer but not normal cells [106]. Using this knowledge, bismuth selenide (Bi_2_Se_3_) nanosheets decorated with chitosan and RGD peptide showed the ability to target α_v_β_3_ integrin on cancer cells, and specific biodistribution of nanosheets was observed to be involved in the suppression of cell growth at tumor sites. Uptake of the Bi_2_Se_3_-chitosan-RGD nanosheets increased prominently in HeLa (cancer) cells, but not in Etc1/E6E7 (normal) cells, resulting in morphological changes of mitochondria and enhanced radiation-induced activation of the p53 signaling pathway involved in cancer cell apoptosis [107]. In a study by Li et al., RAW 264.7 cells (macrophage) were cultured with Bi_2_Se_3_ nanosheets for cellular uptake of the nanosheets, and the macrophages acting as “Trojan horses” (vehicles) were used to transport nanoparticles specifically to tumor tissues [108].

For cell internalization of nanosheets, dimension is an important factor. Nanosheets of different sizes have been applied in cellular research. Small nanosheets were used in experiments of cell transfection or protein sensing in live cells. In a study by Mu et al., GO nanosheets were purchased from Cheaptubes.com (Cheap Tubes Inc., Brattleboro, VT, USA), and nanosheets without coating and protein-coated GO nanosheets were investigated using atomic force microscopy. The averaged diameter and height of GO nanosheets was approximately 0.84 μm and 1.1 nm, respectively [92]. They found that small nanosheets were uptaken by cells through clathrin-mediated endocytosis (CME), and large nanosheets entered cells through CME and phagocytosis [92]. Simulations by Wang et al. suggested that the degree of oxidation, but not the thickness of graphene, is a crucial factor for cell entry [109]. It has been reported that a large size and a higher oxidization degree may be related to stronger cytotoxicity [97,110].

## 6. Cost, Fabrication, and Limitation

Graphene can be produced from natural graphite (flake graphite and microcrystalline graphite). The recent progress in synthetic strategies of 2D nanosheets were summarized by Yang et al., and approaches to the assembly of 2D nanosheets into 3D architectures were demonstrated by Shen et al. [111,112]. Nowadays, graphene sheets are commercially available [113]. For example, GO can be bought from Aladdin^®^ (Shanghai, China) [85]. According to the price list shown by Cheaptubes.com (Brattleboro, VT, USA), single-layer GO could be purchased at a price of $400.00–$450.00 per gram, and the price of few-layer GO is approximately half that. On the other hand, most of the newly developed nanosheets are still commercially unavailable. Graphene is fabricated by several methods, including chemical vapor deposition, micromechanical exfoliation of graphite, and so on [5,114,115,116]. During the process of chemical vapor deposition, a temperature above 1000 °C was required [117]. Ping et al. developed a cost-effective method to prepare graphene at a relative low temperature (300 °C) [118]. Further, a spin-coating method was developed to fabricate graphene and metal oxide nanosheets with a size of approximately 30 mm in 1 min, which also lowers the cost [76]. However, even when the nanosheets are made from the same material, the fabrication processes are important because of cost and biocompatibility evaluation. Kuilla et al. have pointed out that the quality of graphene is an important factor determining its performance [119]. Biological molecules can be adsorbed more easily onto thin graphene nanosheets with fewer layers compared to thicker ones [120]. Small and sharp nanosheets should be internalized easily into cells, and nanomaterials containing impurities may affect the cell uptake mode-of-action and cytotoxicity [12,120,121]. Microsized GO induced stronger inflammation responses compared to nanosized GO [121]. In a study by Yue et al., GO was prepared using a modified Hummers method, and the sheets were separated by using specific sedimentation rates, e.g., 100–200 g and 10,000–30,000 g centrifugal forces for the separation of the GO nanosheets of 2 μm and 350 nm, respectively [121]. Fabrication methods of other types of nanosheets were reviewed by Kurapati et al., and they pointed out that the methods can be summarized as the top-down approach and the bottom-up approach: the former is based on the direct exfoliation of bulk material, and the latter is the process of generating nanosheets via atomic-level control of their composition and structure [122]. In cellular research, nanosheets are not applied as synthesized; additional processes, such as functionalization, modification, and coating, are always necessary (examples shown in Table 1). Further, as mentioned above, an increasing number of nanosheets types are emerging as new biomaterials using conventional medical materials, and these nanosheets could meet specific requirements for applications in cellular research. For example, a nanosheet structure on titanium alloys (Ti6A14V) was formed by a simple NaOH treatment (alkali etching, 10 M ad. NaOH treatment at 30 °C for 24 h) [13].

Currently, there are still limitations to the development and application of nanosheets in cellular research. First, isolation of graphene nanosheets was just realized in 2004, and nanosheets have been used in cellular research for only about 10 years. The understanding of cell–nanosheet interaction is limited with respect to the mechanical and electrical properties of nanosheets [29]. As mentioned above, cells may respond to physical factors such as size and oxidative degree through specific functional proteins, and a high concentration of nanosheets may be toxic to cells [92,110,123]. Therefore, when a new type of nanosheet is developed, large numbers of pre-experiments on cells may be necessary to optimize the fabrication processes. Without any functional improvement, nanosheets may not only influence cell adhesion and spreading, but also induce previously uncharacterized cellular responses [37], which may result in an inflammatory response. Second, regarding their application as scaffolds in tissue engineering, although there have been many types of nanosheets, few sophisticated techniques have been developed for the induction of nanosheets into a three-dimensional structure as an in vivo-like microenvironment. Cells cultured on a solid flat surface as a monolayer may respond to drugs differently from those in three-dimensional culture models [124]. Interaction of a specific functional group of nanosheet with cells is crucial to the cells’ ability to adhere [97]. Although nanosheets have been applied to strengthen the scaffold in tissue engineering, there are no techniques to control the local distribution and alignment of nanosheets within three-dimensional structures. It is expected that there is little diffusion in a solid scaffold, and without precise control of the local distribution, there may be undefined cell–nanosheet interactions. There are still other reasons for precise control. For example, mechanical properties of articular cartilage are different at the surface, middle, and deep layers, and, to regenerate engineered tissue, precise control of mechanical properties is necessary [125]. Further, Bressan et al. pointed out that, because graphene is in the form of a dry powder at some point, there may be a potential health risk through inhalation [86]. Graphene-family nanomaterials may also lead to adsorptive and quenching artifacts in biological assays [86,126].

## 7. Conclusions and Perspectives

Application examples of nanosheets in cellular research are listed in Table 1. This review investigates the effects of several types of nanosheets on cells and the development of fabrication techniques. As a substrate, nanosheets may influence the function of cells, depending on parameters such as the sheet thickness and the mechanical properties of supporting substrate. The surface of nanosheets can be modified to reduce cell cytotoxicity and improve biocompatibility. Coating of nanosheets with specific antibodies makes the nanosheets sensitive to targeted cells. Since nanosheets can interact strongly with DNA or drugs and have a fluorescence-quenching ability, these materials have been extensively applied as a delivery carrier and as a substrate for cell sensing and imaging in cellular research. Further, nanosheets are involved in regulation of stem cell differentiation, acting as a scaffold for cellular organization. Based on the above-mentioned applications, it can be concluded that nanosheets have several important advantages as a biomaterial benefiting cellular research: (1) Compared to nanoparticles, nanosheets offer a large surface area for cell adhesion or construction of flat nanoplatforms by the attachment of various functional molecules or even nanoparticles. Further, nanosheets show some excellent properties, such as highly polarized positive and negative charges; (2) Unlike conventional biomaterials, such as Polydimethylsiloxane (PDMS) membranes at the microscale, although the thickness may be several nanometers, nanosheets of different lengths and widths—from several centimeters to several nanometers—are available according to experimental needs. Further, nanosheets of the appropriate size can be internalized by cells, and flexible nanosheets with adhered cells can be transplanted through injection; (3) Because the internalization of nanosheets differs between normal and cancer cells, there is a trend to employ nanosheets in the research of cancer diagnosis and therapy. Layered nanosheets are also capable of enveloping drugs, forming a drug delivery structure. Further, nanosheets may change in form (e.g., curled shape to flat shape) in response to microenvironment conditions, such as pH. Even so, the following will promote the application of nanosheets to cellular research in the future: (1) A deep understanding of interaction of nanosheets with cells through cell membranes is a prerequisite. Although there is also a trend to develop nanosheets with conventional medical materials, it is necessary to improve the biocompatibility of nanosheets by performing sufficient investigations on cytotoxicity on specific cell types and improving fabrication techniques; (2) Technical breakthrough in harvesting of nanosheets is important for the handling and transplanting of cell/nanosheet constructs. Further, delivery and distribution of nanosheets to targeted cells in a three-dimensional structure, or coupling of nanosheets into a three-dimensional structure, is still an important issue; (3) It will be remarkable to develop design theories and the fabrication techniques for the functionalization of large-scale nanosheets with additional functions for cell culture, such as oxygen permeability and porosity.

## Figures and Tables

**Table 1:** Application examples of nanosheets in cellular research. PEG: polyethylene glycol; hCSF: human corneal stromal fibroblast; PLGA: poly(lactic-*co*-glycolic acid); BP: black phosphorus; MSC: marrow-derived mesenchymal stem cell.

	Materials	Modification/ Functionalization/FABrication	Applications	Effectiveness	Ref.
Cell adhesion	RFL_4_FR	–	Improved adhesion/growth of hCSF cells	Wettability enhancement	[31]
	PLLA	Collagen coating	Adhesion of NIH3T3 cell line		[32]
	Calcium-phosphate nanosheet + titanium	–	Improved adhesion of osteoblast cell	Wettability enhancement	[35]
Delivery substrate	PLGA	Engineered cell monolayer on surface	Injection into subretinal space together with cells	Small size; high flexibility and biodegradability	[25]
	BP	Modification with polyethylene glycol-amine	Drug and dye carrier	Targeted cancer therapy	[52]
	GO	Cell-penetrating peptides	Plasmid transfection into Hela cells	Cytotoxicity reduction and biocompatibility improvement	[54]
Cell capturing	GO	NeutrAvidin (cancer-related biomarker) coating	Capturing of cancer cells: MCF-7, Hs-578T, and PC-3	Sensitive, microfluidic-free, and planar	[64]
	GO	A phase transformation through oxygen clustering	Capturing of Class-II MHC-positive cells	Sensitive, microfluidic-free, and planar	[63]
	GO	Coating with VHH7 and VHH DC 13	Capturing of Class II MHC-eGFP^+^ and CD11b^+^ cells	Effective, rapid, and microfluidic-free	[65]
Cell sensing /imaging	WS_2_	Three-dimensional reconstruction	Sensing of H_2_O_2_ in living RAW 264.7 macrophage cells	Effective in a three-dimensional structure	[77]
	Reduced GO	Decoration with gold nanoparticles and aryldiazonium salts	Sensing of TNF-α secreted by live BV-2 cells	High sensitivity and stability	[78]
	MnO_2_	In combination with fluorescent probe	Tumor cell imaging after cell uptake	Fluorescence off/on switch	[17]
Scaffold elements	Akermanite + boron nitride nanosheets (BNNSs)	Fabrication technique: selective laser sintering system	In vivo-like microenvironment for MG63 osteoblast-like cells	Increased compressive strength and fracture toughness	[82]
	Polymer ethyl acetate solution (poly(d,l-lactic acid) and polystyrene)	Fabrication technique: gravure coating and polymer-based phase separation	Application as a basement membrane for the cell–cell (C2C12 myoblasts) communication	Porous nanosheets	[28]
	PLGA	Self-assemble monolayer of L-cysteine	Transplantation of cells on PLGA	PLGA detachment from substrate in response to a negative electrical potential	[15]
Stem cell differentiation and tissue regeneration	GO	–	Sustaining the self-renewal of mouse embryonic stem cells	A signaling pathway involving integrin	[85]
	GO	–	Differentiation of MSCs to adipogenesis	High affinity of insulin resulting from H-bonding and electrostatic interactions	[87]
	C_3_N_4_ nanosheets	–	Accelerated bone regeneration	Increase of cytosolic Ca^2+^ by photoinduced charge transfer	[88]
Internalization and redistribution of nanosheets and cellular organelles	GO	PEGylation + FITC	Investigation of cellular distribution	Co-localization with F-actin filaments	[90]
	GO or TiO_2_–GO composite	–	Investigation of cytotoxicity on A549 cells	Internalization and entry into the cytoplasm and nucleus	[96]
	Pristine graphene	–	Investigation of the biological effects on murine RAW 264.7 macrophages	Redistribution of pro-apoptotic mitochondrial factors	[99]
	GO	PEGylation	A potential anti-metastatic agent	Impairment of mitochondrial oxidative phosphorylation	[102]
